# Hospital Meetings

**Published:** 1895-06-01

**Authors:** 


### HOSPITAL MEETINGS.

HOSPITAL FOR SICK CHILDREN, GREAT ORMOND STREET.

We have already called attention in these columns to the annual general meeting of hospitals and charities as a golden opportunity more or less wasted, and this view of the matter was certainly borne out by the proceedings at the forty-third annual court of the governors of the above institution, held on Wednesday, May 22nd. It might have been asked, Where were the governors? for those present numbered less than a dozen, all told, and the business transacted was of a purely formal character, chiefly consisting of re-election of officers and votes of thanks. Viscount Gort presided, and the Rev. Dacre Craven, Mr. Arthur Lucas, and Mr. John Murray were amongst those who gathered round the board-room table. The annual report was formally adopted, and a special vote of thanks was accorded to the Duke of Cambridge for his presidency at the recent festival dinner.

Mr. Arthur Lucas announced that the new wing of the building for the reception of cases of scarlet fever, diphtheria, and other infectious diseases, would be open for general inspection on May 31st and June 1st next, when it was hoped by the committee that friends would avail themselves of this invitation to see for themselves the latest addition to the hospital. Mr. Lucas spoke of the difficulty everywhere experienced in finding the necessary funds for the adequate maintenance of London hospitals, but added that he hoped the day was far distant when such maintenance would be thrown upon the rates, a contingency he looked forward to with deep misgiving, and one which he believed meant the stopping of a great tide of sympathy and help.

Very cordial mention was made of the services of the medical staff, and the appointment of Miss Florence Smedley as lady superintendent in place of Miss Close, which had occurred since the last annual meeting, was duly announced.

In view of the fact that money is much needed at the present time to adequately carry on the work of the hospital, we cannot refrain from repeating our conviction that much might be done to arouse and stimulate public interest by a well worked-up annual meeting, and that one of so perfunctory a character as that under discussion is, to say the least, a financial mistake.

MISSION TO DEEP SEA FISHERMEN.

There are perhaps few more popular charitable missions in this country than those which have to do with our sailors and fishermen. It was not, therefore, surprising that the late annual public meeting of the Mission to Deep Sea Fishermen, held at Exeter Hall, was well attended—indeed, that, the hall was fairly packed with an enthusiastic audience. Sir Joseph Savory was in the chair, and in his opening speech referred in warm terms to the good work of the Mission; in regard to its medical work alone no less than 10,000 cases had been treated during the past year. He asked his hearers to reflect upon the enormous number of men engaged in the fishing trade, in providing the thousands of tons of fish which were required every year for consumption in London, and upon the duty of caring for this huge population of the seas, who, but for such missions, would be utterly neglected and unheeded. He appealed to the ladies present for practical help in the shape of those warm woollen and knitted garments which were of such inestimable value to the fisher folk in cold wet weather.

Archdeacon Sinclair moved the following resolution: "That this meeting of subscribers recognises with devout thankfulness to Almighty God the splendid results achieved through the spiritual and philanthropic enterprise carried on by the

Mission to Deep Sea Fishermen during the past twelve years, and, cordially sympathising with the extending work now in progress, pledges itself to an earnest and increased support of the society in the future"—which was supported by the Rev. Prebendary Webb-Peploe; and Mr. Wilfred T. Grenfell, surgeon to the Mission, added some of his personal experiences, his words, coming from the heart of one actually engaged in the difficulties and dangers of the work, appealing vividly to all present. There was afterwards a presentation of good conduct stripes to skippers and members of the Mission vessels' crews by Lady Savory, and a collection made during the singing of a hymn, which was heartily joined in. A good many of the smacksmen were present on the platform. In the evening another meeting was held, when dissolving views were shown to illustrate the society's work in the North Seas and on the coast of Labrador.

We should not forget to mention that books and magazines, new or old, are earnestly asked for, and would be the greatest boon. They may be sent to the secretary, Mr. Francis H. Wood, Bridge House, 181, Queen Victoria Street, London.

### ROYAL HOSPITAL FOR INCURABLES, PUTNEY.

Last week Earl Compton presided at the annual festival dinner of the above institution, which was held at the Albion Tavern. In thanking the governors and committee for having elected him as president, the chairman said he felt he had taken upon himself a great responsibility in accepting the honour, and he was determined that what he might lack in experience should be made up in close attention to the business and interests of the institution. As to the work of the hospital, Lord Compton especially commended the scheme of out-door pensions as one of the best conditions of the charity. These pensioners, incurables, were scattered throughout the country, and by the bounty of £20 a year were enabled to live among their own friends in comfort. He concluded by appealing for further help, and especially for additional annual subscribers. It was afterwards announced by the secretary that the subscriptions and donations amounted to over £2,000.

### CITY ORTHOPÆDIC HOSPITAL.

Festival dinners follow fast one upon the other at this season of the year, until it would seem that the tide of charity in that direction must surely run dry. Amongst others which took place recently must be mentioned that in aid of the City Orthopædic Hospital, held in the Venetian Room of the Holborn Restaurant, over which the Earl of Dudley presided. This hospital was first founded in 1851, and the board of management are just now anxious to collect some £5,000 to enable an additional wing, at present closed for want of funds, to be opened for the reception of cases. Lord Dudley, in proposing success to the charity, remarked that the triumphs in recent years in the department of orthopædic surgery had been exceptionally striking, and many cases were now found to be curable which not so very long ago would have been given up as hopeless. The hospital was entirely dependent upon voluntary contributions, and the staff largely gave their services. During the evening subscriptions amounting to £750 were announced.

### NATIONAL SOCIETY FOR EMPLOYMENT OF EPILEPTICS.

We have taken pleasure from time to time in chronicling the success of the much needed efforts of this excellent society, and in giving some account of the work it is accomplishing at the Colony at Chalfont St. Peter, Bucks. It will be remembered that the foundation stone of the first permanent home was laid last November by Mr. Passmore Edwards, and the report which was presented at the first annual meeting of the society, held at the offices, 12, Buckingham Street, Strand, on Monday last (Mr. E. M. Nicholls presiding), shows that good progress is being made in its erection. The medical report was a most encouraging one, and stated that "the experience of the first half-year from the opening of the colony has been a pleasant surprise even to those who were most sanguine as to the ultimate success of an Epileptic colony." Marked improvement in health of mind and body has been noted, and also a corresponding improvement in the spirits of the colonists and the heartiness with which they work. In all cases, it is said, the fits have become less severe, and for one whole month not a single fit occurred among the eighteen inmates of the homes during their hours of work in the garden, &c. The abundant supply of fresh, wholesome food, in the production of which the inmates have a large share, has, no doubt, proved a most important factor in the excellent general health reported, and the characteristic moodiness and irritability are much less marked than in the early days of the experiment. The feeling of a common interest, and of self-help, is doing much to induce a condition of things which can only act for good upon all the members of this little colony. It is hoped in course of time that as the colony grows, still more may be done in the way of a variety of occupations and employments, and the executive committee are now pleading for funds to enable them to extend their work, and especially to confer the benefits of the colony upon women and children. The society has some very warm friends, and will certainly gain many more as its work becomes more widely known, and no one could visit the home at Chalfont St. Peter without becoming a hearty supporter of a scheme which is proving itself able to do so much for the alleviation of real and undeserved misery.

